# Correlation Between Intracranial Arterial Calcification and Imaging of Cerebral Small Vessel Disease

**DOI:** 10.3389/fneur.2019.00426

**Published:** 2019-05-01

**Authors:** Yuan-Chang Chen, Xiao-Er Wei, Jing Lu, Rui-Hua Qiao, Xue-Feng Shen, Yue-Hua Li

**Affiliations:** Institute of Diagnostic and Interventional Radiology, Shanghai Jiao Tong University Affiliated Sixth People's Hospital, Shanghai, China

**Keywords:** intracranial arterial disease, cerebral small vessel disease, intracranial arterial calcification, magnetic resonance imaging, computed tomography angiography

## Abstract

**Background and Purpose:** Vascular calcification is part of the atherosclerotic process. Intracranial artery calcification is closely associated with cerebral small vessel disease (SVD). The present study aimed to investigate the distribution pattern of intracranial arterial calcification and its association with magnetic resonance imaging (MRI) markers of SVD in patients with acute ischemic cerebrovascular disease.

**Methods:** Two hundred and seventy six consecutive patients with transient ischemic attack (TIA) or acute ischemic stroke who underwent both computed tomography (CT) angiography and MRI were enrolled in this study. Intracranial arterial calcium scores were evaluated using Agatston method. MRI was performed to assess cerebral infarction, white matter hyperintensities (WMHs), lacunes, cerebral microbleeds (CMBs), and enlarged perivascular spaces (EPVSs).

**Results:** Intracranial artery calcification was present in 200 (72.46%) patients, with the highest prevalence in the internal carotid arteries (ICA) (64.8%). The severity of intracranial arterial calcification was associated with the presence of WMHs (*P* = 0.0001), lacunes (*P* = 0.0001), and CMBs (*P* = 0.0001); however, there was no association between calcifications and the presence of EPVSs (*P* = 0.058). The correlation coefficients (r_s_) were 0.350, 0.142, 0.285, and 0.251 for WMHs, EPVSs, lacunes, and CMBs, respectively. The adjusted odds ratios (ORs) of intracranial arterial calcification were: 2.747 for WMH (grade 1–2), 3.422 for WMH (grade 3), 2.902 for lacunes, 2.449 for CMB, 0.88 for EPVS (grade 1), and 0.295 for EPVS (grade 2–4).

**Conclusion:** Intracranial artery calcification is common in patients with ischemic cerebrovascular disease and the intracranial carotid artery is most frequently affected. Intracranial arterial calcifications might be associated with imaging markers of SVD and are highly correlated with WMHs, lacunes, and CMBs. Quantification of calcification on CT provides additional information on the pathophysiology of SVD. Intracranial arterial calcification could act as a potential marker of SVD.

## Introduction

Atherosclerosis is a systemic vascular process that is considered a major cause of cerebrovascular and cardiovascular disease (1). Vascular calcification is part of the atherosclerotic process and may indicate severe stenosis (2). Atherosclerotic calcification occurs in the form of hydroxyapatite deposits that resemble bone mineralization (3). The more advanced stages consist of calcified plaques (1), which can be assessed non-invasively using computed tomography (CT) (4). The association between calcification in different arteries and vascular diseases has been examined previously (5). Vascular calcification on CT is a predictor of coronary heart disease (6) and is a potential marker for clinical stroke (7). Recent evidence suggests that the presence of intracranial arterial calcifications could independently predict the occurrence of a stroke in Caucasian and Asian individuals (2, 8). Despite arterial calcification usually occurring in the atherosclerotic large artery, it has been reported that intracranial arterial calcification is also associated with small vessel disease (SVD) (5, 9–11).

Cerebral SVD is an intrinsic disorder of the small vessels of the brain, including small arteries, arterioles, capillaries, and small veins (12). SVD is a common cause of stroke and dementia, and causes considerable worsening of cognitive function, balance, and gait (12). On brain magnetic resonance imaging (MRI), six closely correlated features are markers of SVD, including recent small subcortical infarct, white matter hyperintensities (WMHs), lacunes, cerebral microbleeds (CMBs), enlarged perivascular spaces (EPVSs), and atrophy (13). Various studies have investigated the associations between atherosclerosis and MRI markers of SVD, and found that intracranial arterial calcification is closely related to SVD (11, 14–18). The pathological changes in vessels seem be correlated with parenchymal alteration (12); therefore, we hypothesized that intracranial arterial calcification might be a risk factor for SVD. The aim of the current study was to investigate the distribution pattern of intracranial arterial calcification and its association with MRI markers of SVD in patients with acute ischemic cerebrovascular disease. We hoped to determine whether cerebral artery calcification could act as a marker of SVD.

## Materials and Methods

### Population

The inclusion criteria were: Patients who underwent transient ischemic attack (TIA) or acute ischemic stroke, who presented within 7 days of symptom onset; were between 18–80 years old; who underwent CT, CT angiography (CTA), and MRI; had no critical medical conditions; no history of head trauma; and no history of tumors. TIA and Stroke were defined according to accepted clinical criteria (19, 20). Between September 2017 and June 2018, 297 consecutive patients visited our hospital within 7 days of symptom onset and were initially considered for inclusion in the present study. All the patients underwent CT immediately when they visited the hospital and underwent MRI within 7 days of the visit. The exclusion criteria were: Patients who did not undergo CT, CTA, and MRI; patients with contraindications for CTA and MRI; those with poor imaging quality; patients with a critical medical condition, head trauma, or tumor. Any patients with other possible sources of white matter hypoattenuation on a chart review—such as multiple sclerosis, acute disseminated encephalomyelitis (ADEM), vasculitis, or connective tissue diseases—were also excluded. Ultimately, 276 patients were enrolled, including 114 (41.3%) females and 162 (58.7%) males. The average age of the patients was 65.5 ± 6.3 years. There were 201 (72.8%) first-ever stroke patients and 75 (27.2%) recurrent stroke patients.

This study was carried out in accordance with the recommendations of institutional guidelines. The protocol was approved by the committee of Shanghai Jiao Tong University Affiliated Sixth People's Hospital institutional review board. All subjects gave written informed consent in accordance with the Declaration of Helsinki. This study adhered to standard biosecurity and institutional safety procedures.

### CT Acquisition and Processing

CT angiography (CTA) was performed using a 128-slice multidetector CT (Definition AS, Siemens Medical Solutions, Forchheim, Germany), with the following parameters: 120 kVp; 150 mA; pitch, 0.601; slice acquisition interval, 0.5 mm; slice thickness, 1 mm; and intravenous administration of 120 mL of iodinated contrast media (Iobrix) at a rate of 3.5–4.0 mL/s under the control of the autoinjection program.

### Degree of Calcification

The calcium score was evaluated on non-enhanced CT. Contrast-enhanced CT was performed to determine the vessel wall to identify whether the high-density region was in the artery (calcification) or out of the artery (bony structure). The calcium score was evaluated using the Agatston method (21) on an offline workstation (Syngo, Siemens, Forchheim, Germany) for further analysis. A calcified plaque was defined as a radiation-attenuating structure with an attenuation above a threshold of 130 Hounsfield units (Hu) in an area of 1 mm^2^. Carotid bulb calcium plaques were identified in a soft tissue window (width 340 Hu, center 43 Hu), and a narrow viewing window (width 1 Hu, center 130 Hu). For quantification, the region-of-interest function was used to measure the area and peak attenuation of the calcified plaques. A score for each calcified plaque was obtained by multiplying the plaque area by a weighted peak attenuation score. The weighted peak attenuation was the product of peak attenuation and a cofactor of 1–4: Cofactor 1 (130–199 Hu), cofactor 2 (200–299 Hu), cofactor 3 (300–399 Hu), and cofactor 4 (400 Hu). For each patient, score was calculated for each intracranial artery, including anterior and posterior circulation arteries. The sum of these gave the total score, which represented the sum of all single calcium plaque scores in the intracranial arteries. Intracranial artery calcium scores were also divided into four severity grades on the basis of coronary artery calcium data: None: Calcium score 0; minimal–mild: Calcium score 1–100; moderate: Calcium score 101–400; severe: Calcium score 400 Agatston units (22).

### MRI Examination and Analysis

MRI examinations were performed using a 3.0T MRI system (MAGNETOM Skyra 3.0T, Siemens, Amberg, Germany). MRI images were obtained parallel to the orbitomeatal line, using the following parameters: Time repetition (TR)/time echo (TE) = 5,120/62 ms, slice thickness = 4 mm, field of view (FOV) = 220 × 220 mm, three different directions of diffusion gradient and two b values (0 and 1,000 mm^2^/s) for diffusion-weighted imaging (DWI); TR/TE = 7,500/81 ms, slice thickness = 4 mm, and FOV = 220 × 220 mm for fluid-attenuated inversion recovery (FLAIR) imaging; TR/TE = 4,730/72 ms, slice thickness = 4 mm, and FOV = 220 × 220 mm for T2-weighted images; and TR/TE = 28/20 ms, slice thickness = 1 mm, and FOV = 220 × 220 mm for susceptibility weighted imaging (SWI). No contrast material was administered.

A recent infarct was defined as a hyperintense area on DWI, with a corresponding reduced signal on the apparent diffusion coefficient image, with or without an increased signal on T2-weighted imaging or FLAIR, which corresponded with a typical vascular territory. Recent small subcortical infarcts were defined as ovoid or rounded lesions with similar signal characteristics to recent infarcts but were small (>3 mm and < 20 mm) in diameter, in the centrum semiovale, internal capsule, basal ganglia, or brainstem, and were carefully distinguished from WMHs (23). Cortical infarcts were defined as infarcts involving cortical adjacent subcortical tissue, or large (>20 mm) subcortical/ striatocapsular lesions (13). Deep and periventricular WMHs were both coded according to the Fazekas scale from 0 to 3, using T2-weighted images and FLAIR (24). Lacunes were defined as ovoid or rounded lesions, small (>3 mm and < 20 mm) in diameter, in the centrum semiovale, internal capsule, basal ganglia, or brainstem, with cerebrospinal fluid (CSF) signal intensity on T2-weighted images and FLAIR, generally with a hyperintense rim on FLAIR and no increased signal on DWI (13). CMBs were defined as small (< 5 mm), homogeneous, round foci of low signal intensity on gradient echo images in the cerebellum, brainstem, basal ganglia, white matter, or cortico-subcortical junction, differentiated from vessel flow voids and mineral depositions in the globi pallidi (13). CMBs were detected based on SWI. EPVSs were defined as small (< 3 mm) punctate (if perpendicular) and linear (if longitudinal to the plane of scan) hyperintensities on T2 images in the centrum semiovale or basal ganglia, and they were rated on a validated semiquantitative scale from 0 to 4 (25). In this study, we only counted CMBs and EPVSs in the basal ganglia because in this region, they seem to be specifically associated with SVD (26, 27).

### Clinical and Laboratory Informations

The patient's data for traditional vascular risk factors (28) and previous strokes were collected. Hypertension was diagnosed as present when a patient had a resting systolic/diastolic blood pressure of ≥140/90 mm Hg in repeated measurements or had been taking blood-pressure-lowering agents. Diabetes mellitus was defined as a fasting blood glucose level of ≥7.0 mmol/L or treatment with oral glucose-lowering medications or insulin. Hyperlipidemia was defined as a total cholesterol level of ≥6.2 mmol/L, a low-density lipoprotein cholesterol level of ≥4.1 mmol/L, or if the patient had been treated with lipid-lowering medication after a diagnosis of hyperlipidemia at admission. Smokers were defined as if they were current smokers or had stopped smoking within 1 year before the index stroke. Previous stroke was defined if the patient had a previous stroke-like symptom combined with an ischemic lesion confirmed by brain imaging, and a history of TIA was excluded. Coronary artery disease was defined as having a history of myocardial infarction, unstable angina, or angiographically confirmed occlusive disease of the coronary artery. Atrial fibrillation in electrocardiography was defined as an irregularly spaced QRS complex (Q, R, and S waves) without a discrete P wave.

Two experienced neurologists, who were blinded to the patient's clinical information, independently reviewed the CT images and graded the degree of intracranial artery calcification. The interobserer agreement of calcification grading was found to be acceptable (*k* = 0.948, *p* < 0.001). Then the presence of WMHs, EPVSs, CMBs, and lacunes was observed independently outside the acute infarct area (based on DWI). The interobserver agreement values for the presence of WMHs, EPVSs, CMBs, and lacunes were 0.944, 0.936, 0.928, and 0.901, respectively (all *p* < 0.05). Any disagreement was resolved by consensus.

### Statistical Analysis

Continuous data were analyzed using the analysis of variance (ANOVA) test. Univariate analysis was performed using the χ^2^ test for categorical data. A Mann–Whitney U test or Kruskal–Wallis test was used to compare the median calcium score among the different severity groups by SVD MRI markers. Spearman rank correlation test was performed to analyze the correlation between the calcium score and each SVD MRI marker. A trend χ^2^ test was used to examine the evidence of association between the degree of cerebral arterial calcification and the severity of the MRI markers of SVD. Univariate and multivariate analysis were performed using logistic regression analysis to investigate the association of intracranial artery calcification (Calcium score >0) with each of the SVD MRI markers. The multivariate analysis was adjusted for age, sex, hypertension, diabetes mellitus, hyperlipidemia, previous stroke, current smoking, atrial fibrillation, prior coronary artery disease, and three other SVD MRI markers. The results were expressed as crude odds ratios (ORs) (univariate analysis) and adjusted ORs (multivariate analysis) together with their 95% confidence intervals (CIs). A *p*-value of < 0.05 was considered significant. The statistical packages SPSS 17.0 (IBM Corp., Armonk, NY, USA) was used for the analysis.

## Results

Among the 276 patients (mean age: 65.5 ± 6.3 years, 41.3% female), any intracranial artery calcification was present in 200 (72.5%) patients. The baseline characteristics by calcification severity (calcium score) are shown in Table 1. Age, hypertension, diabetes mellitus, current smoking, and prior coronary artery disease were significantly different among the different calcification severity groups (*P* = 0.0001, 0.006, 0.01, 0.001, and 0.0001, respectively). The highest prevalence of calcification was seen in the internal carotid arteries (64.8%), followed by the vertebral arteries (30.2%), basilar artery (19.5%), middle cerebral arteries (6.3%), anterior cerebral arteries (1.7%), and posterior cerebral arteries (1.2%).

**Table 1:** Baseline characteristics by the intracranial artery calcium score.

**Calcium Score**	**0**	**1–100**	**101–400**	**400+**	***p*-value**
	**(*n* = 76)**	**(*n* = 65)**	**(*n* = 70)**	**(*n* = 65)**	
**DEMOGRAPHIC DATA**					
Sex, female	29 (38.2%)	26 (40.0%)	29 (41.4%)	30 (46.2%)	0.804
Age, years^*^	59.7 ± 5.7	64.7 ± 4.0	67.8 ± 4.3	70.7 ± 4.9	0.0001
**RISK FACTORS**					
Hypertension^*^	38 (50.0%)	41 (63.1%)	49 (70.0%)	50 (76.9%)	0.006
Diabetes mellitus^*^	18 (23.7%)	22 (33.8%)	30 (42.9%)	32 (49.2%)	0.01
Hyperlipidemia	27 (35.5%)	26 (40.0%)	24 (34.3%)	23 (35.4%)	0.908
Previous stroke	16 (21.1%)	15 (23.1%)	23 (32.9%)	21 (32.3%)	0.262
Current smoking^*^	15 (19.7%)	15 (23.1%)	25 (35.7%)	31 (47.7%)	0.001
Atrial fibrillation	8 (10.5%)	14 (21.5%)	14 (20.0%)	13 (20.0%)	0.281
Prior coronary artery disease^*^	16 (21.1%)	21 (32.3%)	30 (42.9%)	35 (53.8%)	0.0001

* Significant at P < 0.05

In total, the prevalence of WMHs, EPVSs, CMBs, and lacunes were 73.2, 74.3, 54.0, and 45.7%, respectively. Univariate analysis showed that the severity of intracranial artery calcification was associated with the presence of WMHs, lacunes, and CMBs; however, there was no association between calcification and the presence of EPVSs. The median calcium score values were significantly different among the different severity groups in WMHs, lacunes, and CMBs, but not in EPVSs. The correlation coefficients (r_s_) were 0.350, 0.142, 0.285, and 0.251 for WMHs, EPVSs, lacunes, and CMBs, respectively (Table 2). The crude ORs of intracranial artery calcification (Calcium score >0) were as follows: 4.9 (95% CI 2.6–9.2, *p* < 0.0001) for WMH (grade 1–2), 6.6 (95% CI 3.1–14.1, *p* < 0.0001) for WMH (grade 3), 3.5(95% CI 1.9–6.2, *p* < 0.0001) for lacunes, 2.6 (95% CI 1.5–4.5, *p* < 0.001) for CMB, 1.6 (95% CI 0.1–3.0, *p* = 0.118) for EPVS (grade 1), and 1.7 (95% CI 0.8–3.6, *p* = 0.195) for EPVS (grade 2–4). The adjusted ORs of severe intracranial artery calcification were as follows: 2.7 for WMH (grade 1–2), 3.4 for WMH (grade 3), 2.9 for lacunes, 2.4 for CMB, 0.9 for EPVS (grade 1), and 0.3 for EPVS (grade 2–4) (Table 3).

**Table 2:** Comparison of calcification severity according to MRI makers of SVD.

**Calcium score**			**Median value**	***P*-value**	**r_**s**_**	**Category**				
						**0**	**1–100**	**101–400**	**400+**	***P*-value**
WMHs	Grade 0	(*n* = 74)	93.2			40 (54.1%)	12 (16.2%)	11 (14.9%)	11 (14.9%)	
	Grade 1–2	(*n* = 123)	146.7			24 (19.5%)	31 (25.2%)	35 (28.5%)	33 (26.8%)	
	Grade 3	(*n* = 79)	168.1	0.0001	0.350	12 (15.2%)	22 (27.8%)	24 (30.4%)	21 (26.6%)	0.0001
EPVSs	Grade 0	(*n* = 71)	121.0			25 (35.2%)	13 (18.3%)	17 (23.9%)	16 (22.5%)	
	Grade 1	(*n* = 148)	141.1			37 (25.0%)	37 (25.0%)	38 (25.7%)	36 (24.3%)	
	Grade 2–4	(*n* = 57)	153.5	0.058	0.142	14 (24.6%)	15 (26.3%)	15 (26.3%)	13 (22.8%)	0.518
Lacunes	–	(*n* = 150)	117.9			57 (38.0%)	30 (20.0%)	32 (21.3%)	31 (20.7%)	
	+	(*n* = 126)	163.0	0.0001	0.285	19 (15.1%)	35 (27.8%)	38 (30.2%)	34 (27.0%)	0.0001
CMBs	–	(*n* = 127)	117.1			48 (37.8%)	28 (22.0%)	27 (21.3%)	24 (18.9%)	
	+	(*n* = 149)	156.8	0.0001	0.251	28 (18.8%)	37 (24.8%)	43 (28.9%)	41 (27.5%)	0.005

*SVD, small vessel disease; WMH, white matter hyperintensity; CMB, cerebral microbleed; EPVS, enlarge perivascular space; r_s_, correlation coefficient*.

**Table 3:** Intracranial artery calcification in relation to the different MRI markers of SVD.

	**Crude OR (95% CI)**	***P*-value**	**Adjusted OR (95% CI)**	***P*-value**
WMH (grade 1–2)	4.9 (2.5–9.2)	0.0001	2.7 (1.1–6.8)	0.029
WMH (grade 3)	6.6 (3.1–14.1)	0.0001	3.4 (1.1–10.9)	0.037
Lacunes	3.5 (1.9–6.2)	0.0001	2.9 (1.1–7.6)	0.031
CMB	2.6 (1.5–4.5)	0.001	2.4 (1.1–5.7)	0.038
EPVS (grade 1)	1.6 (0.9–3.0)	0.118	0.9 (0.3–2.3)	0.792
EPVS (grade 2–4)	1.7 (0.8–3.6)	0.195	0.3 (0.9–1.0)	0.046

*OR indicated odds ratio per unit increase in the explanatory variable. Adjusted OR was calculated using multivariable analysis, whose entered variables were age, sex, hypertension, diabetes mellitus, hyperlipidemia, previous stroke, current smoking, atrial fibrillation, prior coronary artery disease, and three other SVD MRI markers. SVD, small vessel disease; WMH, white matter hyperintensity; CMB, cerebral microbleed; EPVS, enlarge perivascular space*.

## Discussion

The present study revealed a direct relationship between calcification in the cerebral artery and the presence of MRI-defined markers of SVD in patients with ischemic cerebrovascular disease. Arterial calcification was significantly correlated with the presence of WMHs, lacunes and CMBs, especially for WMHs and lacunes; however, there was no association with presence of EPVSs. To the best of our knowledge, this study was the first to investigate the association between intracranial artery calcification, including anterior and posterior circulation arteries, and four typical MRI imaging factors of SVD using the Agatston Scale, which is a qualitative assessment of calcification.

Our results indicated that arterial calcification correlated with WMHs and lacunes. Our findings are similar to the results of certain previous studies. Chung et al. (11) found that qualitatively graded calcification of the intracranial carotid artery detected by CT was associated with white matter intensities. Nevertheless, the correlation between these two factors in our study was lower than that reported by Chung et al. (11). This might because they only investigated patients with acute ischemic stroke, while we included TIA patients. The latter often show less parenchymal change. Erbay et al. (31) found that high calcium content in intracranial carotid arteries independently correlated with lacunar infarcts. We used the Agatston Scale to quantify the calcification and obtained similar results. Intracranial artery calcification has been reported to be a proxy of systemic arterial stiffness, which could lead limited vasodilation (10). Furthermore, cerebral arterial calcification could impair endothelial function, leading to damaged regulation of vascular tone and the vasodilatory response (29, 30). Therefore, we considered that vasodilation limitation caused by arterial stiffness and endothelium-dependent vasodilation impairment are two potential mechanisms accounting for the WMHs and lacunes, which might lead to hypoperfusion in the small perforating artery territory and cause parenchymal changes (11). However, Erbay et al. (31) also found that the association with WMHs disappeared after adjusting for age. This different result might be caused by the small sample size in their study. Meanwhile, the correlation between atherosclerosis and WMHs is rather complex. Additional studies using a larger patients sample are needed to evaluate this preliminary evidence.

In addition, we found that arterial calcification correlated with CMBs. Chung et al. (14) found an association between carotid arterial calcification and deep CMBs, which was similar to our results. In the present study, we also counted CMBs at the level of the basal ganglia, because deep CMBs were reported to be specifically associated with SVD (26, 27). Some studies indicated that the presence of tight endothelial junctions could protect against hemorrhage, and the blood-brain barrier (BBB) was able to prevent local hemorrhage because of its structural and functional properties (32). Cerebral arterial calcification might impair endothelial function and lead to the increased permeability of the BBB (30). Then, red blood cells could extravasate through endothelial junctions in fragile cerebral small vessel walls, resulting in CMBs (33). Moreover, Saba et al. (34) found that atherosclerotic disease of the carotid artery was associated with the presence of CMBs. This could have results from via a common underlying process in which atherosclerosis might involve carotid arteries as well as the small vessels, which become damaged and allow blood to leak outside the vessels (34). Vascular calcification is part of the atherosclerotic process (2). In this study, we use intracranial arterial calcifications as a proxy for atherosclerosis, and found a similar result.

However, we did not observe a significant correlation between EPVSs and cerebral arterial calcification. The exact pathogenetic mechanisms involved in EPVSs formation remain controversial. Previous studies proposed different mechanisms to explain the association between atherosclerosis and EPVSs, including increased arterial stiffness, intraluminal pressure in small intracranial vessels, chronic cerebral hypoperfusion, or merely a coexistence of cerebral SVD and atherosclerosis (35–38). Del Brutto et al. (38) found a relationship between intracranial atherosclerosis and enlarged basal ganglia perivascular space (BG-PVS) using a qualitative assessment of calcification; however, the mean age of the participants was 71.2 ± 8.4 years. EPVS has been described as an age-related phenomenon (35). In the present study, we investigated the correlations in patients with acute ischemic cerebrovascular disease using enhanced CT scan and quantitative assessment of calcification, and did not obtain a similar result to that of Gutierrez et al. According to our results, we considered the main risk of cerebral arterial calcification was an impaired endothelial function that leads to increased permeability of the BBB (30), which may not play a major role in the formation of EPVSs. Thus, this might indicate the need to further explore the mechanism underlying the pathogenesis of EPVSs.

In our study of patients with acute stroke and TIA, cerebral arterial calcification was highly prevalent (72.5%), and most frequently affected the ICA (64.8%). Previous studies reported different prevalences of cerebral arterial calcification. Among patients with acute cerebrovascular disease, the rates of intracranial and cervical vessel calcifications ranged from 40 to 87% (39). ICA calcification was found in 69% of consecutive Chinese patients who underwent brain CT (40). The high prevalence noted in the present study might have been caused by the high sensitivity of MDCT and the use of Agatston Scale to detect calcification automatically. The distribution pattern of cerebral artery calcification was similar to those seen in the previous studies (40). In this study, we found that the main determinants of intracranial artery calcification were older age, diabetes mellitus, current smoking, and prior coronary heart disease. Intracranial artery calcification has been associated with smoking, hyperlipidemia, history of cardiac disease, ischemic stroke, renal failure, atrial fibrillation, diabetes, hypertension, gender, and age (39). Our findings supported some of the associations previously reported.

Our study had some limitations. It study comprised cross-sectional research of patients using brain CT and MRI screening. The enrolment of patients in the present study population may have introduced bias. Another possible limitation involves the fact that the calcified lesion on CT is merely a part of the complete atherosclerotic plaque. The “soft” part of the plaque was not measured, which is assumed to be most vulnerable and generally does not contain calcium. However, autopsy studies have found that the severity of atherosclerosis is correlated highly with the extent of atherosclerosis across cerebral arteries, specifically in the intracranial vasculature. Consequently, it is likely that if there is more intracranial artery calcification, there are possibly more atherosclerotic changes in the distal cerebral vessels. From this perspective, cerebral arterial calcification would be a marker of total intracranial atherosclerosis (8).

In conclusion, intracranial arterial calcification is common in patients with ischemic cerebrovascular disease and the ICA is the most frequently affected artery. Intracranial arterial calcifications might be associated with MRI-defined markers of SVD and are highly correlated with WMHs, lacunes, and CMBs. Quantification of calcification on CT provides additional information concerning the pathophysiology of SVD. Intracranial artery calcification could act as a potential marker of SVD. Further studies are needed to address the clinical consequences of this correlation.

## Ethics Statement

This study was carried out in accordance with the recommendations of institutional guidelines, committee of Shanghai Jiao Tong University Affiliated Sixth People's Hospital institutional review board with written informed consent from all subjects. All subjects gave written informed consent in accordance with the Declaration of Helsinki. The protocol was approved by the committee of Shanghai Jiao Tong University Affiliated Sixth People's Hospital institutional review board.

## Author Contributions

Y-HL and Y-CC conceived and designed of the study. Y-CC, R-HQ, and X-FS organized the database. X-EW and JL performed the statistical analysis. Y-CC wrote the first draft of the manuscript. Y-HL, Y-CC, X-EW, and JL wrote sections of the manuscript. All authors contributed to manuscript revision, and read and approved the submitted version.

### Conflict of Interest Statement

The authors declare that the research was conducted in the absence of any commercial or financial relationships that could be construed as a potential conflict of interest.
